# Comparing Antidepressant Effects of Psilocybin-Assisted Psychotherapy in Individuals That Were Unmedicated at Initial Screening Versus Individuals Discontinuing Medications for Study Participation: Comparaison des effets antidépresseurs de la psychothérapie assistée par la psilocybine (PAP) chez les personnes non médicamentées à la sélection initiale et les personnes ayant arrêté les médicaments pour participer à l’étude

**DOI:** 10.1177/07067437251328316

**Published:** 2025-03-25

**Authors:** Noah Chisamore, Erica S. Kaczmarek, Zoe Doyle, Danica E. Johnson, Geneva Weiglein, Shakila Meshkat, Ryan M Brudner, Marc G. Blainey, Jeremy Riva-Cambrin, Roger S. McIntyre, Joshua D. Rosenblat

**Affiliations:** 1 Department of Pharmacology, 7938 University of Toronto, Toronto, ON, Canada; 2 654596 Brain and Cognition Discovery Foundation, Toronto, ON, Canada; 3 Institute of Medical Science, 7938 University of Toronto, Toronto, ON, Canada; 4 Department of Psychiatry, 7938 University of Toronto, Toronto, ON, Canada

**Keywords:** depressive disorders, treatment-resistant depression, mood disorders, psilocybin, psilocybin-assisted psychotherapy

## Abstract

**Objective:**

To compare changes in depression, anxiety, and suicidality symptoms after a single 25 mg oral dose of psilocybin between treatment-resistant depression participants not on antidepressants at screening to participants that discontinued antidepressant medications leading up to receiving psilocybin-assisted psychotherapy (PAP).

**Methods:**

Participants (n = 27) received at least one 25 mg dose of psilocybin accompanied by psychotherapy as part of an exploratory analysis from an open-label, randomized, waitlist-controlled clinical trial. The primary outcome of changes in depression symptoms was measured by the Montgomery-Åsberg Depression Rating Scale (MADRS). Secondary outcomes included changes in anxiety symptom severity (Generalized Anxiety Disorder 7-Item [GAD-7]), suicidal ideation (MADRS Item-10), self-reported depression symptoms (Quick Inventory for Depression Symptomology [QIDS-SR]), and intensity of psychedelic experience (Mystical Experience Questionnaire 30-item [MEQ30]). Patients were separated into two groups for analysis; those who were unmedicated at initial screening versus participants that had to taper off antidepressant medications to be eligible for the trial. A mixed analysis of variance was used to evaluate clinical outcomes over time from baseline to 2 months post-dose.

**Results:**

No significant differences were found between medication discontinued (n = 18) and unmedicated at screening (UAS) (n = 9) groups in clinician rated depression (p = 0.759), self-reported depression (p = 0.215), anxiety (p = 0.178), and suicidality (p = 0.882) symptoms over time, with both groups having clinically significant benefits on all outcomes assessed. Both groups also had a similar intensity of psychedelic experience (p = 0.191).

**Conclusion:**

Comparable improvements were observed in depression and anxiety and symptoms between antidepressant discontinued and UAS patients. These findings contrast with and contribute to the growing literature on the effects of medication tapering leading up to PAP. Further clinical research is needed to directly compare efficacy across medication statuses, in addition to evaluating psychedelic effects in individuals continuing antidepressants during PAP.

## Résumé

**Objectif:**

Comparer les changements dans les symptômes de dépression, d’anxiété et de suicidalité après une dose orale unique de 25 mg de psilocybine entre les participants atteints de dépression réfractaire sans aucun antidépresseur au moment de la sélection et les participants sevrés graduellement avant la psilocybine.

**Méthodologie:**

Les participants (n = 27) ont reçu au moins une dose de 25 mg de psilocybine en plus d’une psychothérapie dans le cadre d’une analyse exploratoire d’un essai clinique ouvert randomisé, contrôlé par liste d’attente. Le principal critère d’évaluation – changements dans les symptômes dépressifs – a été mesuré à l’aide de l’échelle *Montgomery-Åsberg* (MADRS). Les critères secondaires étaient les changements de sévérité des symptômes d’anxiété (échelle d’évaluation de l’anxiété généralisée – 7 items [GAD-7]), de pensées suicidaires (MADRS – item 10), des symptômes dépressifs auto-évalués (*Quick Inventory for Depression Symptomatology* [QIDS-SR]) et de l’intensité des expériences psychédéliques (*Mystical Experience Questionnaire* – 30 items [MEQ30]). Les patients ont été divisés en deux groupes pour l’analyse : ceux qui n’étaient pas médicamentés à la sélection initiale et ceux qui devaient arrêter graduellement les antidépresseurs pour être admissibles à l’essai. Une ANOVA mixte a été utilisée pour évaluer les paramètres cliniques entre le début de l’administration et deux mois plus tard.

**Résultats:**

Aucune différence significative n’a été observée entre les groupes (sevré de médicaments [n = 18] et non médicamenté à la sélection [n = 9]) concernant l’évolution des symptômes dépressifs évalués par le clinicien (*p* = 0,759), les symptômes dépressifs auto-évalués (*p* = 0,215), l’anxiété (*p* = 0,178) et la suicidalité (*p* = 0,882), les deux groupes affichant des améliorations cliniquement significatives selon tous les paramètres évalués. L’intensité des expériences psychédéliques était également similaire dans les deux groupes (*p* = 0,191).

**Conclusion:**

Les améliorations des symptômes de dépression et d’anxiété étaient comparables chez les patients ayant arrêté les antidépresseurs et les patients non médicamentés à la sélection. Ces résultats contrastent avec la littérature portant sur les effets du retrait graduel des médicaments jusqu’à la PAP et contribuent à la documentation croissante sur le sujet. Une recherche clinique plus poussée s’impose pour comparer directement l’efficacité selon le statut médicamenteux et évaluer les effets hallucinogènes chez les individus qui poursuivent le traitement antidépresseur durant la PAP.

## Introduction

Treatment-resistant depression (TRD) is classified as a depressive episode that has inadequately responded to at least two trials of antidepressant medication.^1–3^ TRD is highly prevalent, affecting an estimated 30% of patients diagnosed with depression, necessitating the need for novel treatments beyond first-line pharmacotherapies.^1,4^ TRD contributes to significant negative deficits in overall well-being and functioning of patients, while increasing the cost and burden to the healthcare system.^1,4,5^ Those with TRD experience significantly greater risks of relapse of depressive symptoms, suicidality, and all-cause mortality.^6^ There is an urgent need for effective, safe, and novel therapeutic agents for TRD.

Psilocybin is a serotonergic psychedelic derived from the *Psilocybe* genus of mushrooms.^7^ Both psilocybin and its active metabolite psilocin display high-affinity agonism for the 5-hydroxytryptamine (HT) 2A receptor (5-HT2A).^7^ Preliminary evidence suggests that serotonergic psychedelics are well tolerated and alleviate symptoms of depression in combination with psychological support (psilocybin-assisted psychotherapy (PAP)).^8–10^ Prior research has demonstrated that 25 mg of psilocybin is a therapeutic dose for both major depressive disorder (MDD) and TRD.^11–13^

Recently, it was suggested that discontinuing concomitant antidepressants, specifically serotonin and norepinephrine reuptake inhibitors (SSRIs and SNRIs), may attenuate the antidepressant response of PAP.^14^ Typically, participants are required to discontinue and taper off antidepressant pharmacotherapies prior to enrolling in psilocybin clinical trials, particularly antidepressant medications that increase serotonin levels.^11,13–16^ Medication tapering is also conducted due to antidepressant medications increasing serotonin transporter occupancy and potential downregulation of serotonergic receptors that may dampen the therapeutic response to psilocybin.^8,17,18^ There is also the risk of serotonergic toxicity to consider when combining antidepressants and psychedelic treatments that both operate on the serotonergic system.^19^ Additionally, there are concerns that continuing serotonergic antidepressants may diminish the psychedelic effects of psilocybin.^16,20,21^ However, separate findings showed no difference in psychedelic experience between unmedicated and discontinued groups.^14^

Other factors to consider in medication tapering are the adverse effects and worsening depression symptoms that may result. When stopping antidepressants suddenly, patients may experience symptoms of serotonin discontinuation syndrome, including nausea, imbalance, flu-like symptoms, insomnia, and dizziness.^22–24^ In an effort to reduce the severity of discontinuation syndrome, antidepressants should be tapered gradually over several weeks.^25^ However, even a conservative tapering approach can still cause adverse effects that impact patient functioning and overall well-being.^22,25–27^ There is also a risk of relapse towards worsening depression symptoms.^18^ A patient's decision to discontinue antidepressant medications for a clinical trial itself may foster expectations that PAP will significantly improve the participant's depressive symptoms.^28,29^ Although psychotherapy and other supports could help reduce the risk of relapse following antidepressant discontinuation, this risk cannot be completely eliminated.^26^ Overall, more research is needed on how discontinuing antidepressant medications can impact clinical outcomes in PAP research.

In this post-hoc analysis, the aim is to evaluate the efficacy of psilocybin with psychological support and therapy for TRD in unmedicated at screening (UAS) and medication discontinued (MDC) patients. Data from a randomized, waitlist-controlled, open-label clinical trial was used to conduct analyses regarding depression, anxiety, and suicidality symptom outcomes.^13^

## Methods

### Study Design

A randomized, waitlist-controlled clinical trial was conducted at Braxia Health (formerly the Canadian Rapid Treatment Center of Excellence), a community clinic in Mississauga, Ontario, Canada, which specializes in assessing, caring for, and researching persons with treatment-resistant mood disorders. The community Institutional Review Board (Advarra) approved this trial (Pro00056530; BCDF001), and the trial was registered on ClinicalTrials.gov (NCT05029466) prior to recruitment. Verbal and written consent was obtained from participants prior to implementing any study procedures. All participants were assessed by a study psychiatrist via a comprehensive physical and mental health evaluation with a clinical diagnostic assessment. An electrocardiogram, routine blood tests, drug urine toxicology test, pregnancy test (as applicable), blood pressure, heart rate, and physical examination were also conducted for safety and eligibility.

For participants taking contraindicated medications prior to enrollment, the study doctor discussed the risks of medication tapering with the patient and their prescribing physician, with the patient deciding if they wanted to do so to participate in the trial. Based on the study doctor's clinical discretion, participants may be excluded from the study if tapering off of their antidepressant medications was deemed unsafe or not clinically appropriate. Tapering was done over 1 month with a 25% dose reduction every week with flexibility provided the participant had no medication changes for 30 days prior to enrollment.

The Usona Institute provided 25 mg doses of powdered synthetic psilocybin to be combined with 100 mL of water for participants to ingest orally. Participants were randomized to either an immediate or waitlist (2-week delay) treatment arm, in blocks of 10 with an equal allocation ratio to the two comparison groups. Participants had up to three psilocybin sessions with a fixed 25 mg dose. Each dose was accompanied by one preparatory therapy session (1–2 h), a dosing session (6–8 h), and two integration therapy sessions (1–2 h each). Multidisciplinary therapist dyads conducted psychotherapy for the trial, all with a license to practise psychotherapy in Ontario. Therapist dyads—consisting of psychiatrists, social workers, spiritual care therapists, psychotherapists, nurses, and primary care providers—underwent a comprehensive PAP training program specifically designed for the trial. At least one member of the therapist dyad was a licensed medical doctor. This program included extensive pre-reading, didactic teaching, supervised cases, and ongoing peer group supervision. The model of therapy was transtheoretical and based on principles described in previous psilocybin trials.^30,31^ Greater detail about the therapy session procedures can be found in the main published manuscript.^13^

### Participants

The trial included participants aged 18 to 75 years old with a primary diagnosis of MDD or bipolar disorder-II (BD-II) currently experiencing a Major Depressive Episode (MDE) of at least 3-month duration. These criteria were assessed by a study psychiatrist according to DSM-5 criteria (Dr. Joshua Rosenblat), and diagnoses were confirmed using the Mini-International Neuropsychiatric Interview. BD-I participants were excluded based on Health Canada's recommendations, given safety concerns of potentially triggering mania or psychosis. No depression severity cut-off was used for this trial. While BD-II patients were included in the original study, they were not included in this exploratory analysis as they were required to stay on mood stabilizing medications during study enrollment. Eligible candidates were either not currently taking pharmacotherapy for depression at trial screening or were willing to taper off current prohibited medications. A classification of TRD was required for inclusion and was defined as having failed to respond to an adequate dose and duration of at least two guideline-concordant pharmacological treatments for the current MDE, using the Massachusetts General Hospital-Antidepressant Treatment History Questionnaire (MGH-ATRQ). No upper limit was placed on the duration of MDE or number of failed antidepressants. Full eligibility criteria can be found through ClinicalTrials.gov (NCT05029466). Participants were not required to pay for participation, nor were they required to be patients at Braxia's private clinic. All participants were compensated to cover travel and expenses associated with study visits.

Ten weeks after the first psilocybin dosing session, participants could be eligible for a second dose of psilocybin as determined by the study doctor, based on clinical evidence of three factors. These factors were clinical benefits from the prior dose, adequate tolerability, and safety of the prior dose and thirdly, signs or symptoms of relapse of depression lasting at least 2 weeks. Relapse of depression symptoms was assessed based on clinical judgment of the patient's symptom severity with access to all research scales to aid in this assessment. Participants were assessed in person or virtually for all study visits and PAP sessions. Follow-up assessments were conducted for up to 24 weeks, with the option of being conducted virtually. All psilocybin dosing sessions were conducted on-site and psychotherapy sessions were conducted on-site preferably when possible or virtually.

### Outcomes and Data Analysis

The primary outcome of this analysis was the absolute change in depression symptom severity over time as assessed by the clinician-administered Montgomery-Åsberg Depression Rating Scale (MADRS). Secondary outcomes included change in anxiety symptom severity (Generalized Anxiety Disorder 7-Item (GAD-7)), suicidal ideation (Question 10 on the MADRS), and self-report of depression symptoms (Quick Inventory for Depression Symptomology (QIDS-SR)). The Mystical Experiences Questionnaire (MEQ) 30-item was used to evaluate the intensity of psychedelic experiences following psilocybin dosing sessions.^32,33^

### Statistical Analysis

All statistical tests were performed using SPSS version 29 software. A mixed analysis of variance (ANOVA) was conducted to determine if there were statistically significant differences in clinical outcome between MDC and UAS groups from baseline to 2-month post-dosing. Baseline demographic and clinical characteristics were compared between groups using t-tests. Mauchly's test was used to evaluate sphericity and ensure the assumptions for parametric testing were met. When sphericity was violated, a Greenhouse-Geisser correction was applied. Data was assessed for normality using the Shapiro-Wilk test and QQ plots, and the presence of outliers was evaluated with box plots. Levene's and Box's tests were used to assess homogeneity of variances and covariances, respectively. All statistical tests were two-tailed, with alpha being set to 0.05.

Outcomes were only considered up to 2 months when participants became eligible for a second dose to avoid introducing variation in the number of doses as a confound between groups. Missing data was extrapolated by carrying forward the last observation for one participant at 2-week post-dose and six participants at 2-month post-dose. The ANOVA model controlled for several covariates: sex, age at baseline, age of first diagnosis, years with depression, and number of past medication trials. A t-test was also used to compare MEQ scores after the first dose as a measure of psychedelic experience.

## Results

### Clinical Characteristics

In total, 30 patients received at least one 25 mg dose of psilocybin, with 26 being included in this analysis after removing BD-II patients. A participant dropped out before receiving the intervention and another participant withdrew from the study prior to their primary endpoint 2 weeks after dosing. To enroll in this clinical trial, 21 (17 MDD) participants decided to taper off their antidepressant medications under the supervision of a study psychiatrist and their prescribing physician. The remaining nine participants were not on antidepressant or contraindicated medications and did not require a medication washout period. Among those who required medication tapering, the mean number of medications was 2.57.

Demographic and clinical variables were compared between MDC and UAS groups at baseline (Table 1). No significant differences were found between groups for any of the baseline characteristics including any clinical symptoms and previously tried antidepressant medication trials (p > 0.05). A baseline MADRS score of approximately 30 is indicative of moderate severity (MADRS score 20–34) of initial depression symptoms in both groups (Table 2).

**Table 1.** Demographics and Baseline Information Compared Between the Medication Discontinued and Unmedicated at Screening Groups Using an Independent Samples t-Test.

Demographic	Medication discontinued	Unmedicated at screening	p-value
Total (n, %)	17 (65.4)	9 (34.6)	N/A
Sex (female, n, %))	6 (23.1)	3 (11.5)	0.846
Age (years, mean SD)	45.3 (15.20)	45.7 (13.75)	0.876
Age of first diagnosis (mean, SD)	25.6 (11.72)	28.8 (7.49)	0.298
Years with depression (mean, SD)	19.7 (13.46)	16.9 (10.30)	0.463
Past medication trials (n, SD)	10.9 (4.76)	10.1 (5.71)	0.838
Baseline MADRS total	30.06 (6.10)	30.33 (6.69)	0.684
Baseline MADRS-SI	1.94 (1.68)	1.67 (1.73)	0.715
Baseline QIDS-SR	16.35 (2.74)	15.78 (2.95)	0.655
Baseline GAD-7	11.12 (5.86)	10.67 (5.24)	0.954

MADRS=Montgomery-Åsberg Depression Rating Scale; GAD-7=Generalized Anxiety Disorder 7-Item; QIDS-SR=Quick Inventory for Depression Symptomology.

**Table 2.** Mean (and Standard Error) of MADRS, MADRS-SI, QIDS-SR, and GAD-7 Scores Between Medicated Discontinued and Unmedicated at Screening Patients from Baseline to 2 Months Post-Dosing.

	Medication discontinued			Unmedicated at screening		
Timepoint	Baseline	Week 2	Month 2	Baseline	Week 2	Month 2
n	17	16	12	9	9	8
MADRS	30.65 (1.54)	20.25 (2.26)	18.17 (2.28)	30.33 (2.23)	22.67 (4.35)	24.22 (3.69)
QIDS-SR	16.35 (0.66)	13.36 (0.98)	13.17 (1.34)	15.78 (0.98)	9.00 (1.45)	11.89 (1.88)
GAD-7	11.12 (1.42)	8.87 (1.44)	9.75 (2.11)	10.67 (1.75)	6.50 (2.45)	9.88 (2.56)
MADRS-SI	1.94 (0.41)	1.75 (0.47)	1.50 (0.50)	1.67 (0.58)	1.44 (0.67)	1.44 (0.69)

MADRS=Montgomery-Åsberg Depression Rating Scale; GAD-7=Generalized Anxiety Disorder 7-Item; QIDS-SR=Quick Inventory for Depression Symptomology.

Although adverse events (AEs) were frequently reported, 28/30 participants who received the intervention in the original study experienced an AE, they were mostly considered mild to moderate and transient. No serious AEs were reported throughout the study. More in-depth reporting of AEs can be found in the main manuscript for the clinical trial.^13^ Additionally, no significant changes in suicidal ideation were found across the entire study sample.

### Depression Outcomes

**Figure 1.**
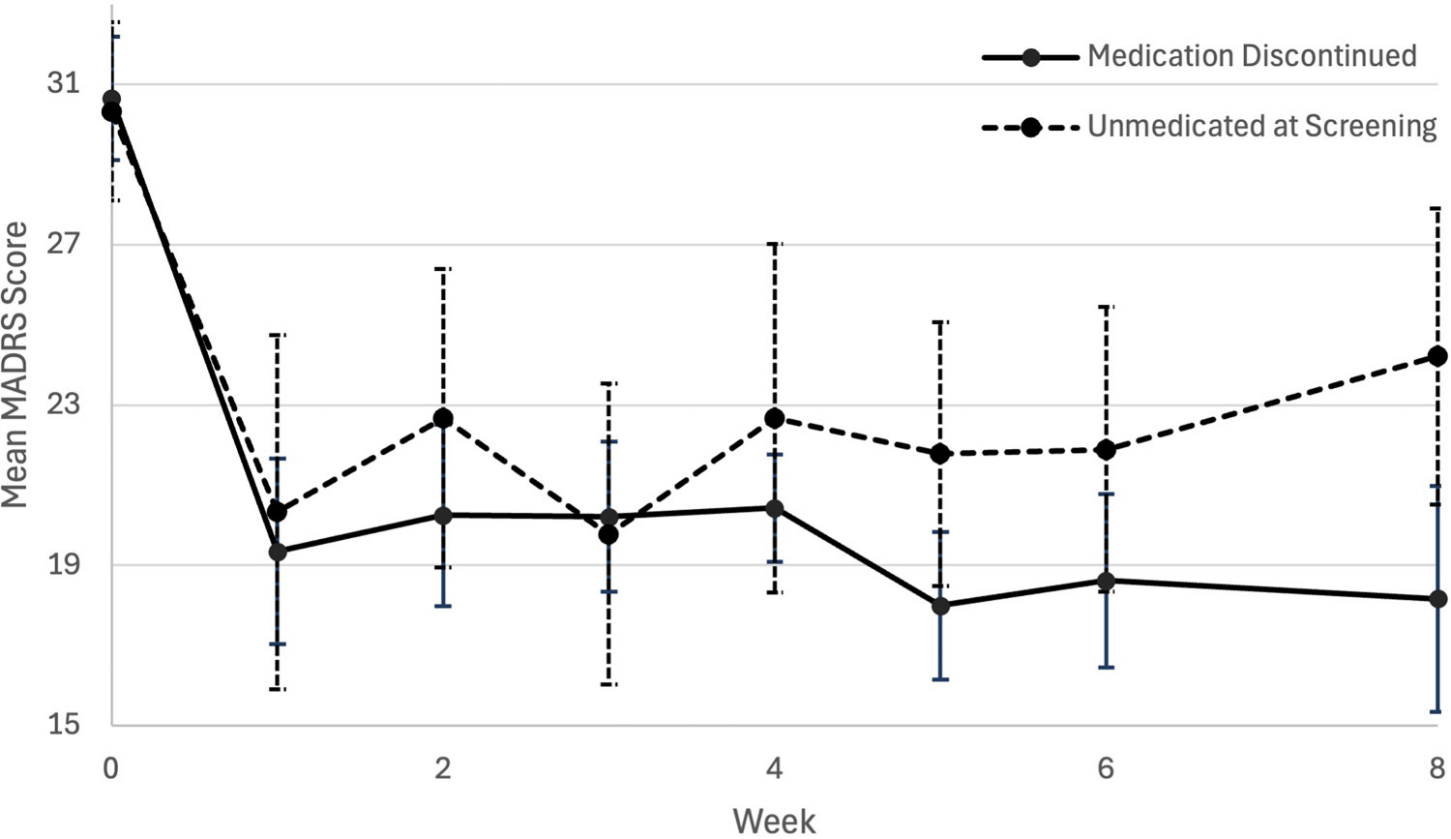
Plot of mean (error bars are standard error) MADRS score from baseline to 2 months (week 8) post-psilocybin dosing between unmedicated at screening and medication discontinued groups. MADRS=Montgomery-Åsberg Depression Rating Scale.

A mixed ANOVA was conducted to compare MADRS scores over the first 2 months of data collection for each participant (Table 2). A single 25 mg dose of psilocybin with psychotherapy was associated with significant differences in MADRS scores over time F (5, 112) = 11.096, p < 0.001, partial η^2 ^= 0.316. There were no significant differences in MADRS scores over time between UAS and MDC discontinued groups F (1. 24) = 0.127, p = 0.724, partial η^2 ^= 0.005. A mean difference in MADRS score over time of 1.191 was found between the UAS and MDC groups (95% CI, −5.693 to 8.075, p = 0.724), with both groups having comparable improvements in depression symptoms at 2-month post-dosing (see Table 1 and Figure 1). Sex at birth, F (1, 27) = 6.768, p = 0.015, partial η^2 ^= 0.200 and baseline (pre-treatment) MADRS scores, F (1,18) = 25.825, p < 0.001, partial η^2 ^= 0.584, were found to be significant predictors of change in MADRS score over time. However, there was no significant interaction effect of baseline MADRS score with medication status group (p = 0.233). It does appear that pre-treatment depression symptom severity may moderate antidepressant efficacy, but this effect does not vary significantly between groups.

A significant treatment effect was also observed in self-reported depressive symptoms over time, as measured by the QIDS-SR16 F (4, 108) = 4.424, p < 0.001, partial η^2 ^= 0.241. There was no significant difference in QIDS-SR16 scores over time between groups, F (1, 23) = 1.269, p = 0.272, partial η^2 ^= 0.052. Only assigned sex at birth, F (1, 23) = 4.858, p = 0.038, partial η^2 ^= 0.174 and baseline MADRS scores, F (1,17) = 44.050, p < 0.001, partial η^2 ^= 0.722, were significant predictors of change in self-reported depressive symptoms.

### Anxiety Symptoms

There were significant improvements in anxiety symptoms as measured by the GAD-7 over time F (2, 51) = 3.950, p = 0.023, partial η^2 ^= 0.141. However, no significant differences were observed between medication groups, F (1, 24) = 1.166, p = 0.291, partial η^2 ^= 0.046, indicating that both the MDC and UAS groups exhibited significant improvements in anxiety symptoms. Both groups had the lowest mean GAD-7 score at 2-week post-dose, and trended towards worsening anxiety symptoms at 2-month post-dosing. Direct comparisons of group differences in mean GAD-7 at 1- and 2-month post-dosing were not statistically significant with mean differences of 2.993 (t (24) = 1.183, p = 0.248) and 1.660 (t (24) = 0.628, p = 0.536, respectively. These results suggest improvements in anxiety symptoms occur in a shorter duration compared to depression symptoms regardless of group.

### Suicidal Ideation

There were no significant changes in MADRS-SI score over time based on dosing, F (5, 116) = 1.259. p = 0.287, partial η^2 ^= 0.050 or between medication groups F (1, 24) = 0.026, p = 0.873, partial η^2 ^= 0.001. As indicated by mean baseline MADRS-SI scores of 1.94 in the MDC group and 1.67 in UAS, mild suicidal ideation was present in this sample prior to treatment. Slight trends towards decreased SI at 2-week post-dosing were observed, but these improvements were not statistically significant. Neither group saw a significant increase in SI, indicating comparable safety with the absence of treatment emergent suicidality.

### Mystical Experiences

**Figure 2.**
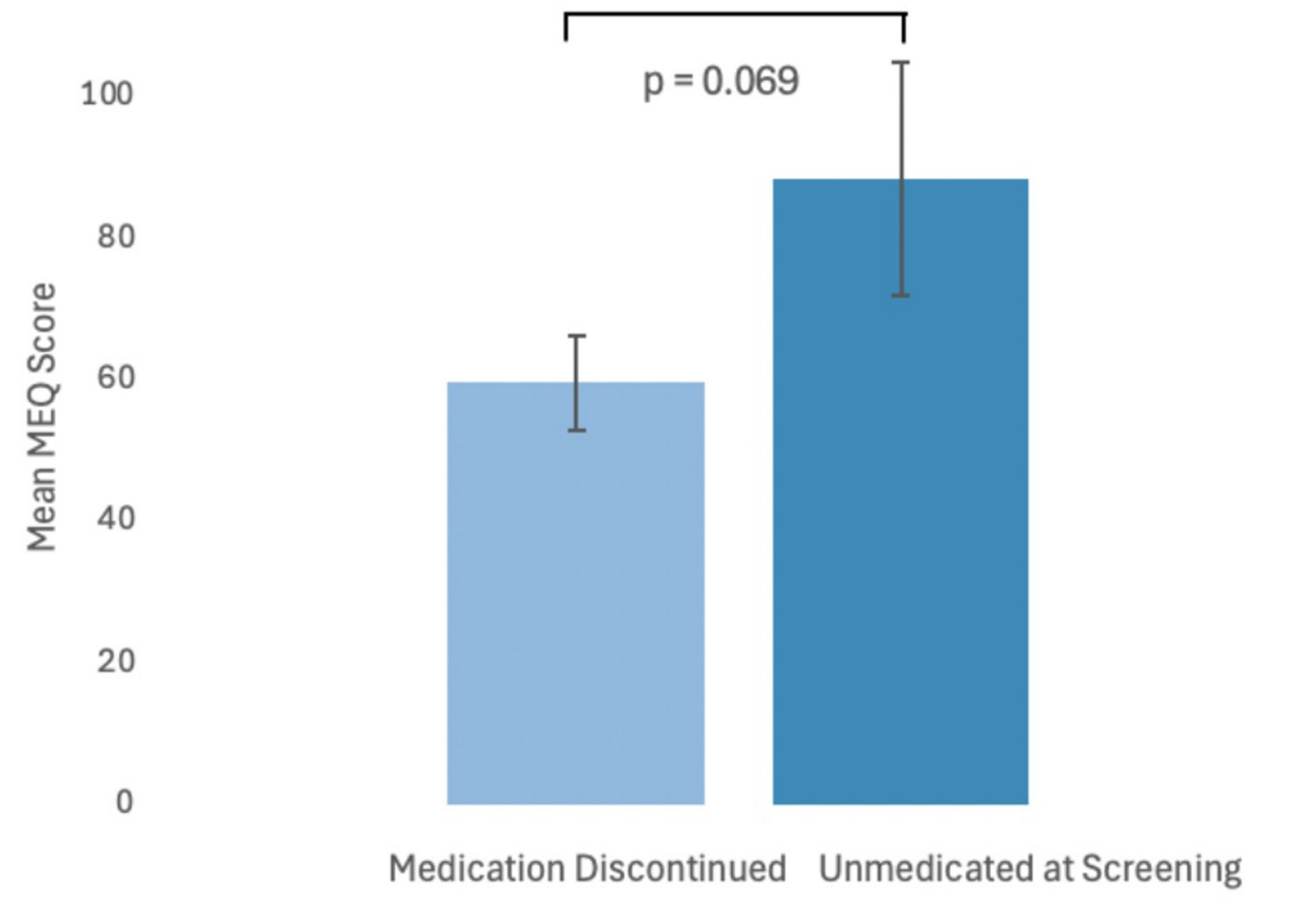
Plot comparing mean intensity (error bars are standard error) of psychedelic experience (MEQ) between medication discontinued and unmedicated at screening groups (p = 0.069). MEQ=Mystical Experience Questionnaire.

The degree of mystical experience as measured by the Mystical Experience Questionnaire 30-item (MEQ30) was not significantly different between UAS (88.56 ± 16.39) and MDC (59.75 ± 6.71) groups at the first psilocybin dose (95% CI, −60.02 to 2.41, t(23) = −1.909, p = 0.069). These results suggest that the intensity of mystical experience was statistically comparable between MDC and UAS participants (Figure 2). MEQ scores were not compared at the second and third doses due to a lack of statistical power.

## Discussion

This exploratory analysis from an open-label clinical trial demonstrated that antidepressant MDC and UAS participants had comparable improvements in depression symptoms following a single dose of psilocybin with supportive psychotherapy. Comparable improvements were also found in self-reported depression symptoms and anxiety symptoms between groups. Neither group experienced significant increases or reductions in suicidal ideation over time. The UAS group had a clinically but not statistically significant greater intensity of psychedelic experience. Our findings contribute to the growing literature on how the efficacy of psilocybin may be impacted by antidepressant medications.

Our results contrast with a recently published article examining the effects of discontinuing serotonergic antidepressants before psilocybin therapy. A post-hoc analysis of an RCT^15^ comparing PAP to escitalopram found that antidepressant discontinuers in the psilocybin group had a reduced treatment effect compared to unmedicated participants.^14^ While these results cannot determine the causation of medication status on PAP efficacy, they suggest the potential for a substantial impact exists.^14^ A greater treatment effect was observed in MDC participants receiving escitalopram, likely as a result of reintroducing an SSRI to those potentially with serotonin withdrawal symptoms.^14,24,26^ It should be acknowledged that there were significant differences between the original trial design and the analyses of Erritzoe et al. Firstly, the data in this analysis stemmed from an open-label, waitlist-controlled trial as opposed to an RCT where escitalopram was used as a control. Erritzoe et al. also analyzed results from MDD patients rather than TRD. Our analysis also has a comparatively smaller sample size and an uneven distribution of MDC and UAS participants (2:1 ratio, respectively, at baseline). The tapered antidepressant medications themselves also differed significantly. In contrast, only SSRIs or SNRIs medications were analyzed in the Erritzoe et al. analysis. The sample for this analysis was required to taper off a more mechanistically diverse range of medications for depression compared to solely serotonergic antidepressants. The reduced prevalence of serotonergic medications among tapering patients could explain why they had a similar response to UAS patients. Overall, the discrepancy in findings between this and the Erritzoe et al. analysis could be attributable to these differences in study design and sample characteristics.

The degree and intensity of psychedelic effects at the first dose were comparable between UAS and MDC participants, though UAS participants had clinically higher MEQ scores. These results support that statistically similar, although not identical from a clinical perspective, strength psychoactive experiences can be produced by a psilocybin dose, even in those requiring a washout period. The analysis from Erritzoe et al. also found comparable psychedelic effects between medication status groups. Furthermore, a study of concomitant SSRIs TRD patients receiving PAP also experienced significant psychedelic effects and improvements in depression symptoms.^16^ Taken together, it appears that continued downregulation of the serotonergic system and 5-HT2A receptor occupancy is not the sole mediator of the psychedelic effects of psilocybin.^14^ There may be slight variation in intensity of psychedelic effects, but PAP dosing sessions can still meet the threshold of an altered state of consciousness irrespective of these variations.

Previous animal research does implicate the serotonergic system in producing psychedelic effects, although other pharmacological factors likely have a role as well.^34–36^ A web-based retrospective survey study of psilocybin users found that serotonergic antidepressant usage was associated with a lower intensity of psychedelic experience compared to non-serotonergic antidepressant usage.^21^ While this study suggests that dampened psychedelic effects could result from more recent antidepressant use, our results and recent clinical research suggest that a profound and therapeutic dosing experience can still occur regardless. Further research is required into what pharmacological factors influence the efficacy and psychedelic experience of PAP. It is currently unknown whether or how exactly the psychedelic experience contributes therapeutic outcomes of PAP.^37^

There are limitations to this analysis that should be addressed. Firstly, this post-hoc analysis is from an open-label trial that lacked a placebo control group and primarily focused on feasibility rather than efficacy. The sample size is small, particularly in the UAS group with only nine participants, reducing the statistical power to detect more nuanced differences in clinical efficacy between groups. This lack of statistical power renders our analysis better described as exploratory rather than being able to display a non-inferiority. Another limitation was that to conduct a mixed ANOVA, some values had to be extrapolated forwards where there was missing data. By looking at outcomes up to only 2 months, the ability to assess longer term efficacy is limited. As well, the study sample had a greater proportion of male than female participants. Considering that depression is more prevalent among females, having a smaller female patient sample reduces the generalizability and representativeness of our analysis. Lastly, mild baseline suicidal ideation limits our ability to compare differences in anti-suicidal effects between medication status groups. Despite these limitations, this analysis is able to contribute to an increasingly important topic on how medication tapering procedures could impact the antidepressant efficacy of PAP.

Future studies should further evaluate the relationship between antidepressant medication tapering and the efficacy of PAP in greater detail. A larger sample size in future clinical research that directly compares the impact of medication tapering on antidepressant efficacy is warranted. Furthermore, it is still being determined if and how the duration of the washout period differentially affects clinical outcomes. As this analysis did not include the four bipolar depression patients from the study, it is important to highlight the lack of research on antidepressant medication discontinuation in PAP for BD-II, where there are likely additional clinical factors to consider in medication tapering.

## Conclusion

This analysis further illustrated the complex and varying impact medication discontinuing has on the antidepressant efficacy of PAP. While we found comparable improvements in depression and anxiety symptoms, and trends toward decreased suicidality between antidepressant MDC and UAS patients, this contrasts with previous research. These findings contribute to the growing literature on medication tapering in psychedelic research and point towards the need for clinical trials designed for direct comparisons of efficacy across medication statuses. It is critical research continues to investigate the best practices for the clinical safety and efficacy of PAP in relation to medication tapering and washout periods.
